# Host Defense Peptides: Dual Antimicrobial and Immunomodulatory Action

**DOI:** 10.3390/ijms222011172

**Published:** 2021-10-16

**Authors:** Matthew Drayton, Julia P. Deisinger, Kevin C. Ludwig, Nigare Raheem, Anna Müller, Tanja Schneider, Suzana K. Straus

**Affiliations:** 1 Department of Chemistry, University of British Columbia, 2036 Main Mall, Vancouver, BC V6T 1Z1, Canada; mdrayton@chem.ubc.ca (M.D.); nigarer@chem.ubc.ca (N.R.); 2 The Centre for Blood Research, University of British Columbia, 2350 Health Sciences Mall, Vancouver, BC V6T 1Z3, Canada; 3 Institute for Pharmaceutical Microbiology, University Hospital Bonn, University of Bonn, Meckenheimer Allee 168, 53115 Bonn, Germany; s6judeis@uni-bonn.de (J.P.D.); kevin.ludwig@uni-bonn.de (K.C.L.); anna.mueller@uni-bonn.de (A.M.); tschneider@uni-bonn.de (T.S.)

**Keywords:** host defense peptides (HDPs), antimicrobial peptides (AMPs), antibacterial, immune modulation, resistance, biocompatibility

## Abstract

The rapid rise of multidrug-resistant (MDR) bacteria has once again caused bacterial infections to become a global health concern. Antimicrobial peptides (AMPs), also known as host defense peptides (HDPs), offer a viable solution to these pathogens due to their diverse mechanisms of actions, which include direct killing as well as immunomodulatory properties (e.g., anti-inflammatory activity). HDPs may hence provide a more robust treatment of bacterial infections. In this review, the advent of and the mechanisms that lead to antibiotic resistance will be described. HDP mechanisms of antibacterial and immunomodulatory action will be presented, with specific examples of how the HDP aurein 2.2 and a few of its derivatives, namely peptide 73 and cG4L73, function. Finally, resistance that may arise from a broader use of HDPs in a clinical setting and methods to improve biocompatibility will be briefly discussed.

## 1. Antibiotic Resistance

The discovery of antibiotic agents has been deemed one of the most impactful advances in modern medicine. Indeed, the administration of penicillin, first discovered by Sir Alexander Fleming in 1928, began what is known as the “era of antibiotics”, where once-deadly infections caused even by a small cut in the skin could be effectively treated [1,2]. The period of rapid antibiotic discovery that occurred shortly thereafter (termed the 1950s “golden era”) contributed to the pivotal role of these agents in medicine and surgery today.

However, specific resistance of the infectious bacteria to these drugs emerged rapidly. In fact, one of the factors initiating the large-scale discovery of antibiotics in the golden era was the need to combat infections by penicillin-resistant bacteria [3,4], which were identified even before the widespread administration of penicillin in World War II [3]. Importantly, β-lactams—a family of chemically modified penicillin derivatives resistant to cleavage by penicillinases, or beta-lactamases—were developed during this period. To date, resistance has been identified for nearly all developed antibiotics [3,4,5]. Even bacteria resistant to vancomycin, a promising antibiotic used for the treatment of methicillin-resistant *Staphylococcus aureus* (MRSA) that displayed very little resistance generation in a laboratory setting, were identified only 7 years after the introduction of the drug into clinical practice in 1972 [4].

Once the traditional approach of modifying existing antibiotics began to lose its efficacy against these multidrug-resistant (MDR) organisms, many large pharmaceutical companies began to abandon antibiotic discovery programs in the 1980s for other avenues promising greater profits [6]. Stricter regulatory barriers by, e.g., the United States Food and Drug Administration (FDA) exacerbated the issue, with reductions in the number of approved antibiotics significantly increasing between 1983 and 2007 meaning even effective antibiotics could be rejected for commercial use [4]. As such, the number of novel antibiotics approved by the FDA each year has greatly decreased [4,7,8]. Promisingly, though, the approval of 9 new drugs between 2018 and 2019, a significant increase from previous years, suggests this may improve in the near future [8].

Nevertheless, the slow development and approval of effective antibiotics means bacterial infections are once again posing a serious threat to global human health. Experts in the field have estimated that, if left unchecked, these antibiotic resistant-pathogens could cause infection-related deaths to rise to levels observed during the Victorian era or cost 10 million lives per year by 2050 [5,9], which some suggest might actually be an underestimate [10]. Further, on a topical note, many are concerned that the recent COVID-19 pandemic may exacerbate resistance and increase this number, as antibiotics are being heavily overprescribed to COVID-19 patients even when only a small proportion (estimated to be 6.9% [11]) also display bacterial co-infection [11,12,13]. As such, it is of utmost importance and urgency to develop novel treatments for these infections, as well as more tightly regulate the current excessive use of antibiotics in healthcare and other fields, such as agriculture, that facilitate resistance generation [3,4].

## 2. Bacterial Cells

**Figure 1:**
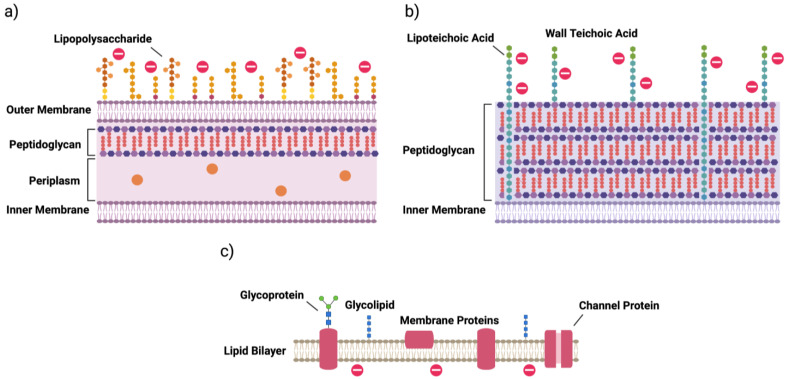
Structures of (**a**) Gram-negative bacterial cell walls; (**b**) Gram-positive bacterial cell walls; and (**c**) mammalian cell membranes.

A key characteristic of an effective antibiotic is its ability to selectively target bacterial cells. Thus, it is important to understand the makeup of these organisms and how they differ from eukaryotic cells. Bacteria are generally categorized into one of two groups based on the composition of their cell walls. These are termed Gram-positive and Gram-negative bacteria, the namesakes of which derive from the bacteriologist Hans Christian Gram, who developed the staining technique used to differentiate between the two [14]. Gram-negative bacteria, in contrast with Gram-positive, contain an outer membrane that is essential for protecting the cell from the environment (Figure 1a) [15]. This outer membrane contains fewer phospholipids than the inner membrane, which themselves are restricted to the inner leaflet, and instead is made up mainly of glycolipids. Of particular note is the glycolipid lipopolysaccharide (LPS), which promotes the secretion of pro-inflammatory cytokines by immune cells [15]. These cytokines are responsible for coordinating the body’s innate defense against infection; however, when released in sudden, large amounts, they can result in sepsis, leading to tissue and organ damage that may be fatal [15,16].

Below the outer membrane of Gram-negative bacteria is a thin, rigid layer of peptidoglycan, a polymer consisting of alternating amino sugars called N-acetylglucosamine and N-acetylmuramic acid [15]. Together, the outer membrane and peptidoglycan wall stabilize the inner membrane, preventing lysis by counterbalancing the high osmotic pressure found within the cell. The periplasm, a viscous space between the inner and outer membranes, is responsible for housing enzymes involved in cell wall maintenance and for sequestering destructive enzymes, such as RNAse [15].

Conversely, Gram-positive bacteria do not contain an outer membrane (Figure 1b). Instead, a much thicker layer of peptidoglycan surrounds the inner membrane [15]. Unique to Gram-positive bacteria, this layer also contains anionic glycopolymers that account for a large proportion of the wall’s mass and play important roles in, among others, membrane stability, membrane function and intercellular interactions [15,17]. One major group of these polymers is teichoic acids, which can be further categorized into lipoteichoic acids and wall teichoic acids (WTAs) depending on their anchoring to lipids of the inner membrane or to peptidoglycan, respectively [17]. These acids impart a generalized negative charge to the surface of the bacteria, a unique feature that is importantly not displayed by mammalian cells (Figure 1c) [18]. Gram-negative bacteria likewise have an overall negative charge due to the phosphate groups present in LPS [19].

## 3. Mechanisms of Antibiotic Resistance

The unique physiological characteristics of bacterial cells can be taken advantage of in the development of antibiotics. In particular, there are five major targets conventionally exploited by antibiotics: the bacterial cell membrane, the peptidoglycan cell wall, nucleic acid synthesis, protein synthesis and metabolic pathways [20,21].

However, as previously mentioned, many bacteria have quickly developed methods to resist the action of conventional antibiotics. Resistance can be intrinsic—i.e., arising from the inherent structural features of a bacterial species, such as efflux pumps that can remove drugs from the cytosol, absent targeting structures and differences in cytoplasmic membrane structures (especially between Gram-positive and Gram-negative bacteria, as discussed earlier); or acquired or developed, either through the acquisition of genetic material from other bacterial species (horizontal gene transfer, HGT) or by mutations in their own chromosomal DNA [22,23]. In fact, the discovery of this genetic mechanism of acquired resistance, first posited in the mid-1950s, completely transformed the field of microbiology and resulted in a much better understanding of the generation and treatment of drug-resistant pathogens [3].

**Figure 2:**
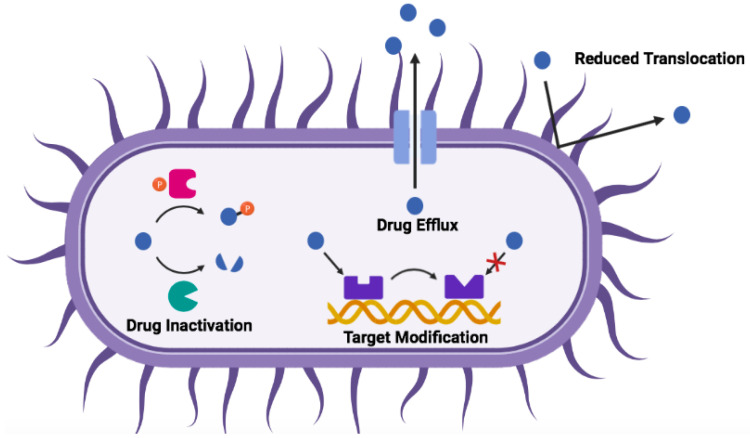
Mechanisms of acquired antibiotic resistance by bacteria. Drug represented as a dark blue sphere.

In general, three main mechanisms of acquired antibiotic resistance have been identified: 1. reduction of intracellular antibiotic concentration via drug efflux and/or decreased membrane penetration; 2. modification of antibiotic targets, either genetically or by post-translational modification; and 3. inactivation of the antibiotic itself by degradation (e.g., hydrolysis) or by biochemical modification (e.g., acetylation and phosphorylation) [22,23]. These mechanisms are summarized in Figure 2.

Due to the limited availability of small molecule antibiotics and their restricted mechanisms of action and activity spectra, there has been great interest in identifying novel antibiotic alternatives for the treatment of existing and emerging MDR bacteria [24]. In recent years, the development of novel antibiotics has exhibited a shift towards the discovery of novel bacterial targets (e.g., new binding sites in bacterial ribosomes, the cell wall, metabolic pathways, etc.) and towards the development of non-traditional approaches to combat infections (e.g., antivirulent, adjunctive, preventative, microbiota-modulating and immunomodulating strategies) [6,24,25]. Interestingly, a large fraction of the agents currently in preclinical development focus on pathogen-specific treatment, which has not generally been prevalent in antibiotic history [6]. One particular promising family of compounds that has garnered much interest as both direct acting and immunomodulating therapies against infection is host defense peptides (HDPs), discussed below.

## 4. Host Defense Peptides

Antimicrobial peptides (AMPs), now more commonly referred to as HDPs, have been identified as key compounds in the host defense systems of virtually all organisms across the three domains of life. In addition to displaying activity against a broad spectrum of bacteria and other pathogenic species [26,27], many HDPs also favorably modulate the host’s immune and inflammatory responses to infection [28,29]. Moreover, HDPs have been associated with slower resistance generation compared to conventional antibiotics [30,31] (more on this in Section 4.3) and have also been found to be effective against biofilm-associated bacteria [24,28,32,33]. These properties highlight clear advantages of HDPs over conventional small-molecule antibiotics, but their success in being translated into clinical practice has been low due to a number of inherent biocompatibility shortcomings, as discussed in Section 4.3.

### 4.1. Structures and Classes

In nature, HDPs are produced both ribosomally and nonribosomally, with the majority of the latter being synthesized by bacterial species [34]. HDPs synthesized by ribosomal translation of mRNA are found more widely, from bacteria to higher order life forms, and have been found to play critical roles in the innate immunity of these organisms. In humans and other mammals, many ribosomal HDPs are stored within immune cells, specifically in the granules of neutrophils, where they can be released locally at sites of infection and inflammation; or they are released in skin and mucosal secretions [34]. Their production is also often tightly regulated, with many being expressed as inactive precursors and later activated by proteolytic cleavage [34]. Expression can be constitutive (e.g., in neutrophils, as discussed), or induced by the presence of pathogen-associated molecular patterns (PAMPs) or cytokines during, e.g., inflammation and infection [34].

Generally speaking, HDPs are short polypeptide sequences ranging from 12–50 amino acids with an overall positive charge (generally ≥ +2) and high hydrophobic character (typically 30–50%) [34,35]. As such, they often possess a large number of tryptophan, lysine and arginine residues. These structural features are responsible for the antimicrobial activity of the compounds: the positive charge enables targeting of bacteria via electrostatic interactions with negatively charged bacterial membranes (see Section 2), while the hydrophobicity facilitates interactions with the phospholipid membrane, which can result in disruption of its integrity or can allow for translocation of the peptide into the cytosol where it can target intracellular processes [34].

A common classification system of HDPs utilizes the peptide’s secondary structure—that is, their propensity to form α-helical, β-sheet or random-coil/extended structures, the first two of which are most common in nature [34,36]. In general, α-helical HDPs are unstructured in solution but form amphiphilic structures when in contact with a biological membrane, whereas β-sheet peptides are more structured in solution due to the presence of disulphide bonds, leading to smaller conformational changes upon interaction with lipid membranes. Even though extended HDPs often possess no secondary structure, they also fold into amphiphilic structures upon membrane interaction and often contain a high number of proline and histidine residues in addition to arginine and tryptophan [34].

### 4.2. Mechanisms of Action for HDPs

Facilitated by their amphiphilic and cationic structures, many HDPs directly destroy bacteria through initial interactions with the bacterial membrane [34]. This key electrostatic interaction forms between the cationic amino acids in the HDP and the negatively charged components of bacterial membranes, such as anionic phospholipids (phosphatidylglycerol, cardiolipin and phosphatidylserine) found in both Gram-positive and Gram-negative bacterial membranes (Figure 1a,b); and teichoic acids and LPS present in the cell wall and outer membrane of Gram-positive and Gram-negative bacteria, respectively [34]. In contrast, mammalian cell membranes (Figure 1c) possess mainly zwitterionic phospholipids, with negatively charged phospholipids facing the cytoplasm in the inner leaflet of the membrane, if present at all. This difference imparts some selectivity onto the HDP between bacterial and mammalian cells. Interestingly, the loss of this asymmetry in inner and outer leaflets in cancer cells that results in negatively charged phosphatidylserine being found on the outer leaflet is partially responsible for the anticancer activity of some HDPs [36,37]. In addition to electrostatic interactions, certain HDPs, such as nisin and mesenterecin, also interact with the bacterial membrane through receptor-mediated interactions [36,38,39].

**Figure 3:**
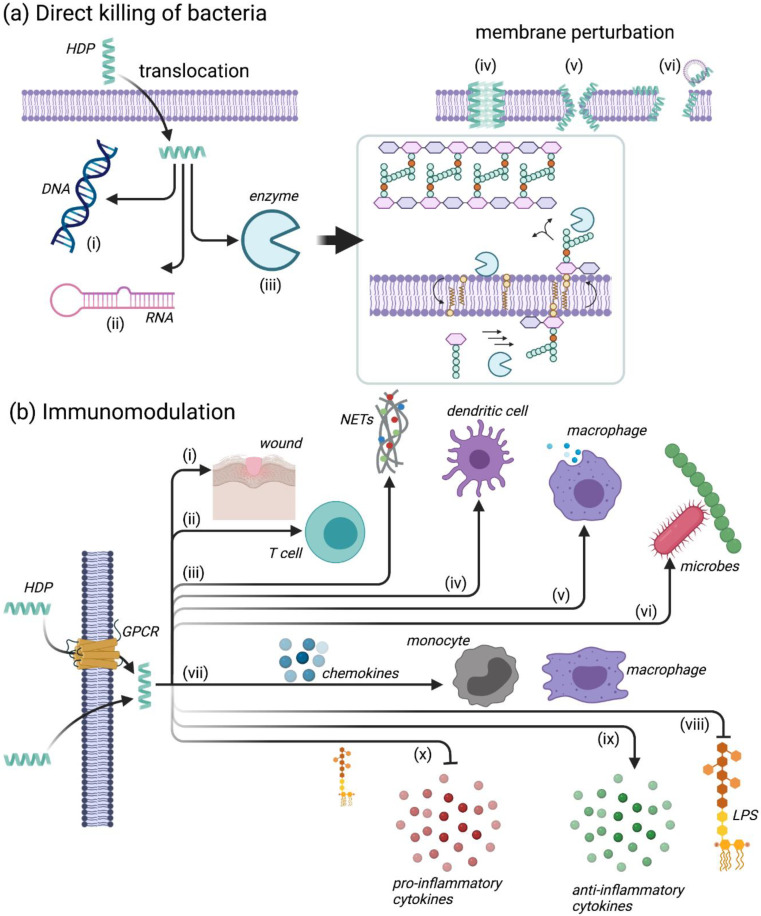
Mechanisms of action of HDPs: (**a**) direct killing of the bacteria, (**b**) immunomodulation. In (**a**), as indicated on the left, HDPs can translocate through the bacterial membrane to reach inner targets, such as (i) DNA, (ii) RNA or (iii) interfere with biosynthetic reactions by binding to proteins or substrates. The inset schematically shows peptidoglycan biosynthesis in *S. aureus*. HDPs can interfere at different points in this pathway. In (**a**), on the right, direct killing through membrane perturbation is shown. The specific mechanisms here are: (iv) barrel stave, (v) toroidal or (vi) carpet or detergent-like pore formation, which lead to cell lysis. In (**b**), intracellular uptake occurs either through G protein-coupled receptors (GPCRs) (yellow) or direct translocation [40]. Once inside, HDPs can function in a myriad of ways: (i) wound healing, (ii) recruitment and polarization of T cells, (iii) promotion of neutrophil extracellular trap (NET) release, (iv) differentiation of dendritic cells, (v) phagocytosis, (vi) modulation of the host microbiome, (vii) induction of chemokines (blue), which leads to the recruitment of monocytes (grey) and macrophages (purple), (viii) neutralization of lipopolysaccharide (LPS), (ix) induction of anti-inflammatory cytokines and (x) suppression of LPS-induced pro-inflammatory cytokines.

The initial interaction with the bacterial membrane is imperative for the direct killing of bacteria by HDPs, which occurs through physical perturbation of the membrane itself and/or through disruption of intracellular processes (e.g., DNA/RNA synthesis, protein synthesis and folding, enzymatic activity, cell wall synthesis, etc.) after translocation through the membrane (Figure 3a) [34]. Once in contact with the membrane, the HDPs form amphiphilic structures (if not present already, as discussed in Section 4.1)—the cationic domains of the peptide interact with the hydrophilic/negatively charged phospholipid head groups, while the hydrophobic domains associate with the hydrophobic fatty acid tails of the lipid bilayer core [34]. Once a sufficient peptide concentration is reached, the HDPs self-assemble at the bacterial membrane, causing membrane permeability either through pore formation (as in the “barrel-stave” and “toroidal pore” models) or through formation of micelles via detergent-like effects (as in the “carpet” model) [34,36]. This membrane permeabilization ultimately leads to leakage of ions and metabolites, which causes depolarization of the transmembrane potential resulting in impaired membrane function (e.g., osmotic regulation) that eventually leads to membrane rupture and lysis [34]. It can also allow for translocation of HDPs into the cytoplasm for intracellular targeting, as previously mentioned. Details pertaining to these mechanisms have been reviewed extensively [32,36,40,41,42,43] and the reader can consult these reviews for further details. Overall, many HDPs likely function through multiple complementary actions, which may be partially responsible for the minimal resistance generated by bacteria toward the compounds [27,34].

In addition to direct bacterial action, many HDPs have been shown to regulate a broad range of immunomodulatory activities that can enhance the host’s response to infection (Figure 3b). These activities include suppression of proinflammatory cytokines and anti-endotoxin activity, which can prevent abnormal and harmful inflammatory conditions (as present in, e.g., sepsis); stimulation of chemotaxis; and immune cell differentiation and activation, which facilitates clearance of bacteria by the host [29,34,36]. Some of these mechanisms will be discussed in detail in the subsections below.

#### 4.2.1. Leukocyte Recruitment

One of the most important mechanisms by which HDPs modulate the immune system is through the enhanced recruitment of leukocytes, i.e., neutrophils, macrophages, mast cells and T cells (Figure 3b(ii,vii)), by the induction of chemokine release [44,45,46,47,48,49,50]. This specific function requires the involvement of several cellular receptors, such as chemokine receptors (e.g., CCR6, CCR2), GPCRs and Toll-like receptors (TLRs) [48]. The HDP LL-37, as well as a number of human β defensins (HBDs) can also function as chemoattractants [29,51,52]; HBD3 specifically has been shown to activate monocytes via TLR1- and TLR2-mediated signaling [53].

#### 4.2.2. Modulation of Neutrophil Function

Alternatively, HDPs can influence the function of neutrophils by stimulating the secretion of neutrophil chemokines, such as interleukin-8 (IL-8) and (growth-regulated oncogene)-α (Gro-α or CXCL1) [54], or the release of neutrophil extracellular traps (NETs) (Figure 3b(iii)). For instance, the HDP LL-37 has been shown to induce the release of neutrophil antimicrobial granule components, including four different human α-defensins, namely human neutrophil peptide 1 (HNP1), HNP2, HNP3 and HNP4 [55]. LL-37 can in addition promote NET formation and consequently help fend off viral infections [56], as well as other pathogens.

#### 4.2.3. Regulation of Inflammation

Some HDPs can act as pro-inflammatory or anti-inflammatory molecules, thereby regulating the inflammation balance and promoting immune homeostasis [40]. They can enhance pro-inflammatory responses to nucleic acids [57] or modulate inflammation through the induction of anti-inflammatory cytokines or the suppression of LPS-induced pro-inflammatory cytokines (Figure 3b(ix,x) [45,58,59]. IDR-1018 has been shown to enhance anti-inflammatory functions while also preserving pro-inflammatory activities needed for the resolution of infection, by driving macrophage differentiation towards an intermediate M1–M2 phenotype [58].

#### 4.2.4. Additional Immunomodulatory Functions

In addition to the functions described above, HDPs have also been implicated in the recruitment of antigen-presenting cells such as monocytes [60], macrophages [61] and dendritic cells [62]. They can also promote phagocytosis [63] and modulate the microbiome [64]. For additional detailed examples, the reader is invited to consult the excellent review by Mookherjee et al. [40] and references therein.

#### 4.2.5. Illustration of the Diversity in HDP Function: From a Natural Peptide to Synthetic Analogues

In order to illustrate how HDP function is related to sequence, we will briefly discuss three related peptides, starting from a natural peptide obtained from amphibians [65].

The aurein peptides are HDPs secreted on the skin of Australian southern bell frogs *Litoria aurea* and *Litoria raniformis* [66]. These cationic HDPs form five aurein families, ranging from short and active peptides (Families 1–3) to longer, typically inactive peptides (Families 4 and 5), which can be further categorized into subfamilies based on length and sequence similarity, denoted by an additional number (e.g., aurein 1.2). Families 1–3 have been particularly well studied, displaying broad-spectrum activity with particularly high potency against Gram-positive bacteria [66].

**Figure 4:**
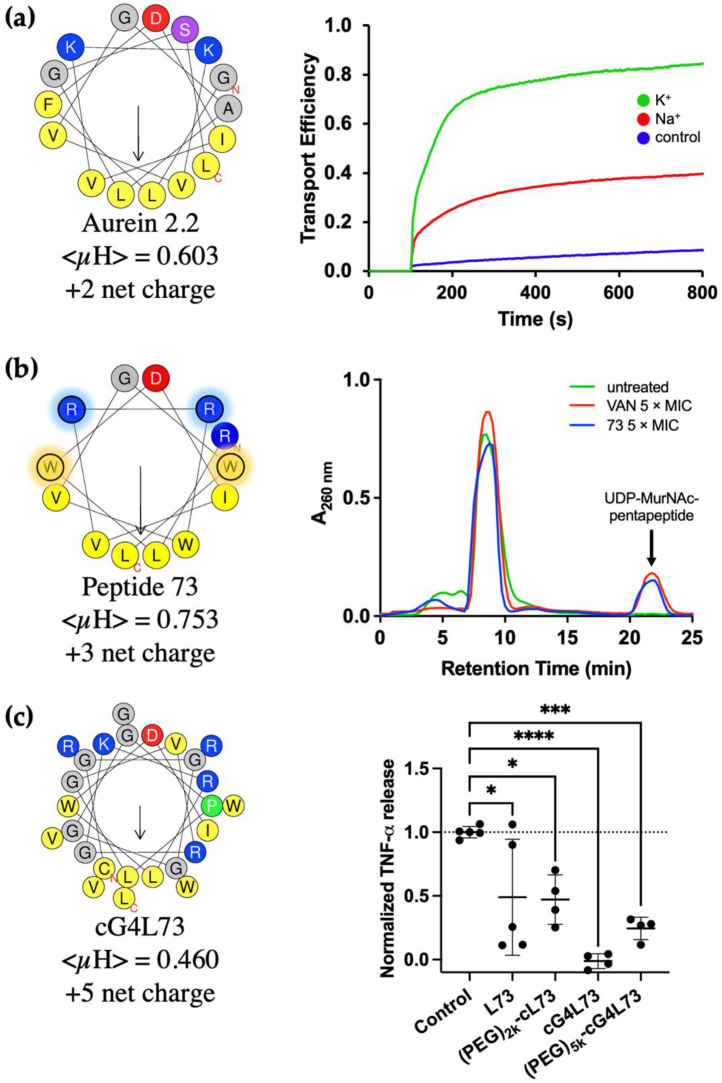
Helical wheel representations (left) of (**a**) aurein 2.2, (**b**) peptide 73 and (**c**) cG4L73. Wheels are generated using https://heliquest.ipmc.cnrs.fr/cgi-bin/ComputParams.py and assume that the peptides adopt a completely α-helical conformation. The mean hydrophobic moment (<μH>) is also given, as is the net charge (note: all the peptides have an amidated C-terminus). On the right is representative data, which illustrates the mechanism of action of each of the peptides: (**a**) aurein 2.2 causes selective leakage of potassium ions, as measured using a pyranine-based in vitro ion translocation assay; (**b**) peptide 73 affects peptidoglycan biosynthesis, leading to an accumulation of UDP-MurNAc-peptapeptide, similar to vancomycin (VAN); (**c**) cG4L73 and its PEG conjugate result in a significant reduction in TNF-α release by LPS-stimulated macrophages (* *p* ≤ 0.05; *** *p* ≤ 0.001; **** *p* ≤ 0.0001 by one-way ANOVA with Dunnett’s multiple comparisons test).

A number of studies [66,67,68,69,70] have shown that aurein 2.2 (Figure 4a), an HDP consisting of 16 amino acids with a net charge of +2 and an amidated C-terminus, functions by forming toroidal pores (Figure 3a(v)) and causing selective leakage of potassium, magnesium and iron. This was initially shown by biophysical studies [67]: (i) circular dichroism (CD) and nuclear magnetic resonance (NMR) were used to show that aurein 2.2 (and its close relative aurein 2.3) forms α-helical structures upon contact with the membrane; (ii) differential scanning calorimetry (DSC) and ^31^P solid-state NMR were used to show that aurein 2.2 and variants [68,69] perturb the membrane bilayer by forming pores, as also evidenced from oriented CD experiments. Using proteomic profiling and a number of assays to determine ion leakage [70] (including the pyranine-based in vitro ion translocation measurements shown on the right in Figure 4a), the membrane perturbation mechanism of aurein 2.2 was further characterized. The fact that aurein 2.2 functions by creating pores is clearly evident when comparing the ion translocation measurements obtained for this peptide relative to others, such as, e.g., daptomycin, which does not function by forming pores [71] (and hence gives results similar to the control seen in Figure 4a [72]). Overall, killing of bacteria results from the disruption of the transmembrane potential of the bacterial membranes by aurein 2.2. Furthermore, the N-terminus of aurein 2.2 was deemed to be important for function, because truncation of aurein 2.2 by three residues from the C-terminus (aurein 2.2-Δ3) was shown not to hinder its antimicrobial activity [70,73].

Starting from aurein 2.2-Δ3, a number of more active peptides were derived from a library, in which the basic and hydrophobic residues of the original peptide were substituted with arginine and tryptophan, respectively [74]. These amino acids were chosen as they have been shown to facilitate electrostatic interactions between the HDP and the bacterial membrane (in the case of arginine) and to mediate peptide-lipid interactions (in the case of tryptophan) [75,76]. Furthermore, both amino acids have been shown to improve peptide-membrane interactions by the formation of cation-π interactions [76,77]. Two peptides, designated peptide 73 (Figure 4b) and 77, displayed improved bactericidal activity against *S. aureus*, even though they possessed less α-helicity than aurein 2.2 in model membranes and caused less membrane perturbation as determined by membrane depolarization and ion leakage [66]. Treatment with the peptides resulted in accumulation of UDP-N-acetylmuramic acid (MurNAc) pentapeptide in the cytoplasm indicating that membrane-bound peptidoglycan biosynthesis steps (i.e., lipid II biosynthesis, transglycosylation/transpeptidation or recycling of the bactoprenyl carrier lipid) are inhibited by 73 and 77 (Figure 4b). Likewise, both peptides induced expression of the *liaI-lux* bioreporter, which is regulated by the cell envelope stress-sensing two-component system LiaRS in *Bacillus subtilis* [78], confirming the previous findings. Additional experiments are required to narrow down the specific reaction affected and to identify the molecular target of 73 and 77. Altogether, the results suggested that peptides 73 and 77 likely function by permeabilizing through the cell membrane and interacting with other targets, such as those involved in cell wall biosynthesis (Figure 3a(iii)), as other arginine- and tryptophan-rich peptides do [79,80,81,82,83].

The sequence of peptide 73 was further modified, leading to the development of peptides such as cL73 and cG4L73 (Figure 4c). These derivatives contain a cysteine residue at position 1, followed by a linker sequence that can be cleaved by matrix metalloproteinases (MMPs). In the case of cG4L73, four glycine residues are positioned between the cysteine and the linker sequence. These peptides were designed to be conjugated to biocompatible polymers such as polyethylene glycol (PEG) or hyperbranched polyglycerol (HPG) (see Section 4.3). Interestingly, cG4L73 and its PEG conjugate both show immunomodulatory activity as probed by the reduction of LPS-induced pro-inflammatory cytokine TNF-α by macrophage-like cells derived from a human monocytic cell line (Figure 4c, right). This was determined by measuring TNF-α release after 24 h cell co-treatment with LPS alongside peptide or conjugate. Further investigations on how cG4L73 and its PEG conjugate might modulate pro- and anti-inflammatory cytokines (i.e., whether these compounds simply bind to cytokines or directly modulate macrophage M1 and M2 phenotype differentiation [58]) are underway.

Overall, HDPs are thought to provide a more robust treatment of infection through combined antibacterial activity and immune system modulation. This multimodal function of HDPs makes them interesting compounds for the treatment of MDR bacterial infections.

### 4.3. Shortcomings of HDPs and Mitigation Strategies

Even though HDPs show tremendous promise, their use in the clinic to date is limited [84,85]. Firstly, HDPs are inherently susceptible to protease degradation due to the l-amino acids that make up their structure, severely limiting their bioavailability and circulation time when administered to the body (due to, in particular, the presence of blood and stomach proteases) [86]. Furthermore, their small size results in quick removal from the body by kidney filtration and the reticuloendothelial system [34]. Secondly, many HDPs display cytotoxicity towards host cells, which can result in systemic toxicity [87]. This is thought to be largely due to the hydrophobic faces of the peptides (as indicated by the mean hydrophobic moment, <μH>, Figure 4), a common characteristic often required for antimicrobial activity, which can enable insertion into mammalian cell membranes. Fourthly, because of their charged nature, HDP activity can be compromised by the presence of salt at physiological concentrations [88]. Finally, although HDPs are touted as giving rise to little bacterial resistance [36,89] (most likely due to their relatively limited use in the clinic), examples of resistance mechanisms have been reported [30,40,90,91]. Specifically, bacteria can evade HDPs in manners that are analogous to those illustrated in Figure 2 for antibiotics—in other words, resistance arises in broad terms by (i) degradation of the HDPs, (ii) sequestration, in particular in biofilms, (iii) physico-chemical modifications of the molecules encountered on the path of HDP entry into the bacterial cell, or (iv) efflux pumps (Table 1). However, studies [30,92] suggest that the listed modifications only confer moderate levels of resistance and are relatively non-specific.

Toxicity and low efficacy of HDPs result in non-superiority over conventional antibiotics and have been reported repeatedly as reasons for failure in Phase III clinical trials [24]. Indeed, the majority of peptides being tested clinically are for topical application, where concerns regarding degradation and widespread toxicity are lessened [24,31]. To minimize these disadvantages, HDP sequences have been studied in the presence of, e.g., red blood cells to take into account physiological salt conditions [88,93]. In addition, small molecule HDP mimetics have been investigated [94,95]. Finally, a variety of chemical modifications and delivery vehicles have been harnessed. These are reviewed extensively in a number of excellent publications [96,97] and will only be mentioned briefly here.

In terms of chemical modifications, isomerization [36,98,99]—that is, replacing one or more of the l-amino acids with d-amino acids, cyclization [100,101,102,103,104], lipidation [105,106] or using peptidomimetic approaches [107,108,109,110,111,112] are common strategies. These alterations often offer a benefit to the HDP by reducing protease degradation. A number of drug delivery vehicles showing potential for HDP formulation include lipid encapsulations [113,114], metal nanoparticles [115,116,117,118], synthetic polymers [74,119,120,121,122,123] and natural biomolecules, such as polysaccharides [124,125,126,127,128], polypeptides [129,130], antibodies [131] and DNA nanostructures [132,133]. These encapsulation or conjugation approaches mainly serve to alleviate toxicity issues.

The improvements in biocompatibility, along with the multimodal functions of HDPs [32,36,40], i.e., their ability to follow multiple paths illustrated in Figure 3 in parallel, and the large number of peptide sequences available [32], will ensure that resistance remains moderate and non-specific. This will in turn ensure that HDPs remain effective antimicrobial and immunomodulatory agents for longer periods of time.

## 5. Conclusions

If left unchecked, the continued rise in multidrug-resistant bacteria will cause infection-related deaths to rise to levels not experienced in generations, where even small injuries might once again be fatal. HDPs, a diverse family of antimicrobial agents produced by virtually all domains of life, present a promising solution to these pathogens due to their high antibacterial activity and, in some cases, auxiliary immunomodulatory properties that potentially provide a more robust response to infection. Indeed, examples were given here of how related peptides, namely the natural aurein 2.2 and its synthetic derivatives peptide 73 and cG4L73, can display a multitude of functions. Additionally, many new approaches are being developed to improve the biocompatibility of HDPs, such as chemical modifications or delivery vehicles. Using a combination of these techniques to improve the limitations of HDPs and considering the vast number of peptide sequences available, it is foreseeable that HDPs will become efficacious therapeutics for the future treatment of multidrug-resistant pathogens, as evidenced by a continued growth in the number of reported clinical studies [2,40,134] (for an up-to-date listing of clinical trials, the website https://clinicaltrials.gov/ct2/home can be consulted).

## Figures and Tables

**Table 1:** Resistance Mechanisms to HDPs for Gram-negative and Gram-positive pathogens. For extensive details on the mechanisms, the reader is invited to consult the excellent reviews of Mookherjee et al. [40] and Joo et al. [30].

Mechanism	Gram-Negative	Gram-Positive
protease degradation	metalloproteinases (e.g., ZapA, ZmpA, ZmpB); aspartate proteases (e.g., OmpT, PgtE, Pla)	metalloproteinases (e.g., aureolysin, SepA); serine endopeptidases (e.g., V8); cysteine proteases (e.g., SpeB)
sequestration	extracellular proteins; biofilm matrix (e.g., alginate, polysialic acid)	extracellular proteins; biofilm matrix (e.g., poly-N-acetyl glucosamine; poly-gamma-glutamic acid)
surface modification	lipid A phosphate modification; lipid A acylation; O-antigen of LPS	d-alanylation of teichoic acids (TA, Figure 1); l-rhamnosylation of WTA; lipid II modification
membrane modification	phosphatidyl glycerol (PG) acylation	PG amino-acylation
efflux pumps	RND family	ABC transporters

## Data Availability

Data supporting previously unreported results can obtained from the corresponding author upon request.
